# Observing charge separation in nanoantennas via ultrafast point-projection electron microscopy

**DOI:** 10.1038/s41377-018-0054-5

**Published:** 2018-08-22

**Authors:** Jan Vogelsang, Germann Hergert, Dong Wang, Petra Groß, Christoph Lienau

**Affiliations:** 1 Carl von Ossietzky Universität, Institut für Physik and Center of Interface Science, 26129 Oldenburg, Niedersachsen Germany; 2 TU Ilmenau, Institut für Werkstofftechnik und Institut für Mikro- und Nanotechnologien, 98693 Ilmenau, Thüringen Germany; 3 Carl von Ossietzky Universität, Forschungszentrum Neurosensorik, 26129 Oldenburg, Niedersachsen Germany; 4 Present Address: Department of Physics, Lund University, Box 118, 221 00 Lund, Sweden

## Abstract

Observing the motion of electrons on their natural nanometer length and femtosecond time scales is a fundamental goal of and an open challenge for contemporary ultrafast science^1–5^. At present, optical techniques and electron microscopy mostly provide either ultrahigh temporal or spatial resolution, and microscopy techniques with combined space-time resolution require further development^6–11^. In this study, we create an ultrafast electron source via plasmon nanofocusing on a sharp gold taper and implement this source in an ultrafast point-projection electron microscope. This source is used in an optical pump—electron probe experiment to study ultrafast photoemissions from a nanometer-sized plasmonic antenna^12–15^. We probe the real space motion of the photoemitted electrons with a 20-nm spatial resolution and a 25-fs time resolution and reveal the deflection of probe electrons by residual holes in the metal. This is a step toward time-resolved microscopy of electronic motion in nanostructures.

The light-induced separation of charge carriers is one of the most fundamental processes in nature. It forms the basis for a vast class of electron transfer reactions in donor-acceptor or light-harvesting complexes^1,4,5^ as well as for a multitude of technological applications, for example, photocathodes^12,16^ and -diodes,^6,17^ and solar cells^3,18,19^. In recent years, nanostructures have become increasingly important for enhancing charge separation, for example, in photovoltaic devices^20,21^, and, in particular, for higher harmonic generation from solids^22^ and ultrafast electron microscopy (UEM)^8–11,23,24^. In UEM, metallic nanotips, for example, driven by strongly enhanced local optical fields have emerged as a new and versatile class of nanoscale electron sources^12–15,25^. In all these structures, the local light-induced birth of charge carriers is intimately connected to an ultrafast real space motion of the photogenerated electron and hole wave packets.

These transport phenomena typically occur at ~10-fs time and ~10-nm length scales. As such, their direct visualization inherently requires ultrafast microscopy techniques with nanometer resolution. Despite recent progress in developing such methods^2,10,11,23,26,27^, the required spatio-temporal resolution and measurement sensitivity are still challenging to obtain. Ultrafast optical techniques provide attosecond time resolution but are inherently diffraction-limited. High photon energy XUV or X-ray spectroscopies can in principle improve this resolution, but they lack the sensitivity to probe dynamics in single nanostructures. In contrast, time-resolved electron microscopies can reach few-nm resolution but are limited to a time resolution of hundreds of fs^28^. More specifically, point-projection microscopes feature shorter propagation distances than conventional electron microscopes but are currently still limited to time resolutions of 100 fs or more by dispersion^11,29^. Fs-photoelectron emission microscopy (PEEM) has been successfully used to image local electric fields at surfaces with few tens of fs and few tens of nm resolution^27,30,31^ and can probe the dynamics of, e.g., plasmons^32^ or excitons^33^ in nanostructures. Generally, PEEM probes how photoelectrons are released from a nanostructure by transient local electric fields. As such, it is, to a certain extent, complementary to point-projection microscopy, which senses the effects of local optical excitations of nanostructures on the dynamics of ultrafast probe electron pulses. Here, we generate such probe pulses by using nanofocusing of surface plasmons on a conical gold taper to induce photoemission from the nanometer-sized apex of the taper. This source is implemented in a point-projection electron microscope (UPEM) and provides a combined spatio-temporal resolution of 20 nm and 25 fs. We use this microscope to directly track the motion of electrons that are photoreleased from the hot spot of a single plasmonic nanoantenna and see how they separate from the positive charges that are left behind in the metal.

In our microscope, we generate ultrashort electron pulses by nanofocusing^15,34–37^ femtosecond surface plasmon polariton (SPP) pulses on a sharp gold nanotaper (Fig. 1a). Few-cycle laser pulses at a wavelength of 1.8 µm with a duration of 15 fs^38^ are focused onto the taper shaft, launching SPPs via a grain boundary at a distance of 80 µm from the taper apex acting as a localized electron emitter. At this wavelength, SPP losses are low, resulting in long-distance plasmon propagation and nanofocusing to an ~ 15-nm-sized focus at the very apex of the monocrystalline gold taper (Fig. 1b)^39^. This nanofocusing is so efficient that high local SPP fields with amplitudes of up to 10 V/nm are generated. These are sufficiently high to release approximately one electron per pulse from a sub-10 nm apex region in a fifth-order photoemission process. The high nonlinearity of this emission efficiently restricts photoemission to the very apex region and effectively creates a free-standing nanometer-sized electron source with a sub-10 fs pulse duration^15^. A characterization of the time structure of the SPP field is provided in the Supplementary Information.

**Fig. 1:**
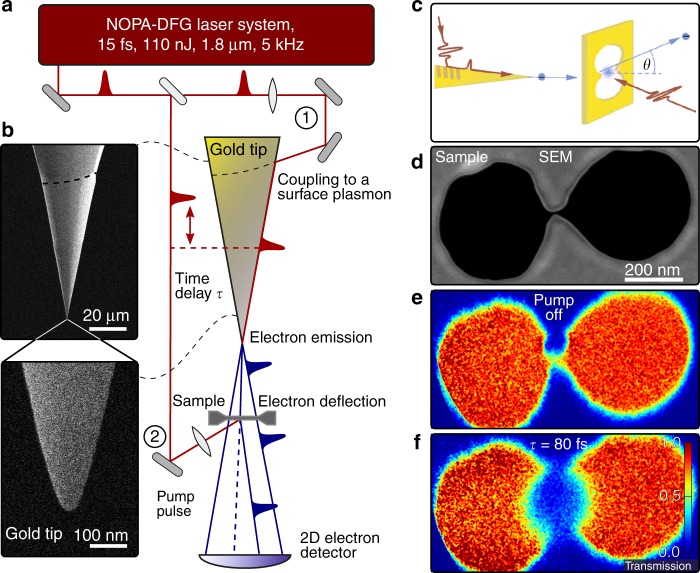
Ultrafast point-projection electron microscopy (UPEM). **a** Schematic of the UPEM setup. **b** Scanning electron microscope (SEM) images of the gold taper used as the electron emitter. **c** UPEM imaging of plasmon-enhanced photoemission. Ultrashort electron probe pulses generated by plasmonic nanofocusing are deflected off a cloud of electrons photoreleased from the gap of a gold nanoantenna. **d** SEM image of a double-nanohole antenna in a 30-nm-thick free-standing gold film. **e** Point-projection image recorded in the absence of a pump laser, mapping the shape of the double-hole nanoantenna. **f** The transient point-projection image recorded 80 fs after illuminating the sample with a femtosecond laser pulse is drastically different: the photoreleased electrons cause a local reduction in probe electron transmission in the region around the antenna gap

This free-standing electron source delivers the probe pulses in our UPEM. In earlier implementations of time-resolved point-projection microscopes^11,40,41^, direct illumination of the laser apex has been used to trigger photoemission. The intense diffraction-limited laser spot used for photoemission has typical diameters of a few microns, and thus, undesired excitation of the sample can only be prevented by limiting the emitter-sample separation to at least a few (tens of) microns. This inherently restricts both the spatial (~ 100 nm)^11,37,40^ and temporal resolution(~ 100 fs)^11,41^ of point-projection microscopy. In contrast, the nanofocused electron source uses evanescent SPP fields to drive photoemission and thus provides the critical advantage of permitting ultrasmall emitter-sample separations.

In our UPEM, the emitted electrons are accelerated toward the sample by a 60 V bias, reducing their relative kinetic energy spread. The incident divergent electron beam is diffracted off the sample. An image of the interference of transmitted and diffracted waves, magnified by the ratio between the detector-emitter (75 mm) and sample-emitter (2700 nm) distance, is recorded on a microchannel plate detector. In static experiments, a similar design already resulted in sub-nm resolution holographic imaging^42^. Under our conditions, the de Broglie wavelength of the incident electrons (~ 0.15 nm) is much smaller than the sample thickness (30 nm). This leads to multiple scattering of the electrons on the sample wall, and phase variations between the scattering events cancel interferences on the detector. The image can thus be explained in the ray tracing limit^10^. The short emitter-sample distance allows us to operate the microscope at high magnification (× 30,000) and, most importantly, effectively suppresses any temporal spreading of the electrons prior to the interaction with the sample. This is the key for advancing the time resolution of electron microscopy in the present experiment.

Here, we use this microscope to study the ultrafast dynamics of photoemitted electrons from a single plasmonic nanoantenna in an experimental configuration that is schematically depicted in Fig. 1c. We design a plasmonic nanoresonator by milling two adjacent holes with a hole diameter of 400 nm in a 30 nm-thin free-standing polycrystalline gold film (Fig. 1d). A small, ~ 30 nm-wide channel connecting the two holes transforms the structure into a nanogap antenna. We make use of the field enhancement in the gap region to induce localized electron photoemission. For this, we illuminate the back side of the antenna with a second time-shifted replica of the 15 fs laser pulse at 1.8 µm. For linearly polarized excitation along the antenna arms, photoemission is induced at a peak electric field strength of 0.6 V/nm. In our experiments, these electrons cannot reach the detector because they are blocked by a 40 V sample-detector bias. The field amplitude of the pump laser is so weak that it does not induce photoemission from the taper apex.

This now allows us to measure background-free point-projection images of the nanoantenna. Hence, any apparent changes in the transient UPEM images are a direct consequence of the interaction of the probing electrons with the optically excited nanoantenna. In the absence of a pump laser, the UPEM image reveals a spatially homogeneous transmission of the probe electrons through the transparent regions of the antenna (Fig. 1e). For a fixed time delay of 80 fs between the optical pump and electron probe, the transmission is largely reduced in a sharply confined region around the channel gap (Fig. 1f). This blocking can be understood as the deflection of the electron probe beam by the cloud of low-energy electrons that is photoemitted from the gap of the antenna^29,43,44^. By changing the time delay between the optical pump and electron probe, we can now create a movie of how this charge cloud evolves in space and time.

Figure 2a shows a time sequence of UPEM images of the relevant gap region. Here, time zero *τ* = 0 fs denotes the coincidence of the optical and electron pulses in the sample plane. For negative time delays, the geometric shape of the nanoantenna is imaged with a resolution of ~ 20 nm, as in the case of a blocked pump laser (Fig. 2b). Around time zero, a reduction of transmission in the central gap region occurs, and the image becomes slightly blurred (Fig. 2c). An almost circular blocking region quickly emerges around the antenna gap. Its diameter expands in time and reaches a value of 200 nm at 80 fs. For longer time delays, the diameter further increases, but now, the rim of the blocking region disappears, and the central region becomes partially transparent again. For *τ* > 150 fs, blurred images of the nanoresonator re-emerge until, at *τ* > 300 fs, the images are virtually indistinguishable from those recorded without the pump. Time zero was deduced as the 50% drop in electron transmission observed for probe electrons passing close to the upper rim of the nanogap (blue curve in Fig. 2d). At all other probe positions, the propagation of the photoreleased electrons away from the nanogap rim results in a delayed drop in transmission (Fig. 2d).

**Fig. 2:**
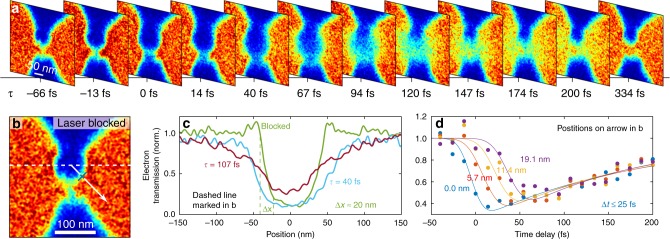
Dynamics of photoemission from the gap of a single plasmonic nanoantenna. **a** Series of transient UPEM images recorded for different time delays _*τ*_ between the laser excitation pulses and probe electrons. UPEM images of the central gap region are displayed, and the electron transmission is color-coded from 0 to 1 using the color bar shown in Fig. 1f. The photoelectrons propagate away from the antenna gap, resulting in a transient local reduction in electron transmission vanishing within 300 fs. **b** UPEM image recorded without laser excitation. **c** Cross cut along the dashed white line shown in **b** at different delay times, giving a spatial resolution of 20 nm. **d** Transmission signal as a function of time at four equidistant positions along the white arrow in **b**. The symbols are the measured electron transmission, and the solid curves represent a phenomenological transport model (see the Supplementary Information). The electron signal decreases from 90 to 10% of its dynamic range within 25 fs, giving the upper limit of the temporal resolution *Δt*

We determine the temporal resolution of our microscope by evaluating the change in transmission at a position close to the upper rim of the gap antenna, marked with a blue tick on the white arrow in Fig. 2b. The transmission dynamics are shown in Fig. 2d (blue circles) and reveal a decrease in transmission within 25 fs (10–90% criterion). This places an upper bound on the time resolution of our UPEM. The reduction in electron transmission vanishes on a 100-fs time scale. The effect of the distance between the gap antenna and electron probe on the dynamics is shown by the additional curves in Fig. 2d. A 6 nm increase in the gap-probe distance (red circles) results in a sizeable time shift of the onset of electron deflection by ~ 11 fs. Looking at the dynamics for a 19 nm gap-probe distance (violet circles), the time shift is increased to ~ 40 fs. At this spatial position, the probe electrons thus see the electrons that are released from the antenna rim around time zero only after a time delay of ~ 40 fs. This sizeable time delay results from the electron propagation from the emission spot to the probe spot. This shows that, already at this small gap-probe distance, photoemission from the gap antenna does not immediately result in a deflection of the probe beam. Instead, this requires a finite propagation of the released electrons from the rim of the gap to the probe position, i.e., a finite separation of the photoemitted electrons from the metal surface. A phenomenological model for this propagation effect is discussed below. Two conclusions can immediately be inferred. First, the deflection of the probe electrons is only sensitive to charges within a distance of < 15 nm from the probe electron trajectory because the probe electrons passing at some distance from the rim are not immediately affected by those electrons that are emitted near the rim. The delayed onset in probe deflection reflects the propagation of photoemitted charges from the rim into the interaction region of the probe electrons. Second, the ratio between the time shift and gap-probe distance provides a direct measure for the speed of propagation of the fastest electrons in the photoreleased cloud. We estimate a speed of 0.5 nm/fs or c_0_/600^44^. This corresponds to a kinetic energy of 0.7 eV, close to our photon energy. Upon a further increase in the gap-probe distance, the observed time delay increases linearly, confirming the picture of a ballistic propagation of the fastest electrons at c_0_/600. In such a ballistic transport picture, we would expect that the dip in transmission vanishes as soon as the electron cloud moves out of the probe volume. The finite persistence of the dip for ~ 100 fs therefore points to a broad distribution of kinetic energies, i.e., propagation speeds, of the released electrons.

To analyze the photoemission process in further detail, we present difference images between the electron transmission at finite time delays and that at a delay of −66 fs, recorded with probe electrons arriving well before the laser pump (Fig. 3a). These data have been taken at a slightly increased pump field strength of 0.7 V/nm. The images show, color-coded in blue, the space-time dynamics of the drop in transmission owing to photoemission from the nanoantenna. The spreading of the released electron cloud and its vanishing within 300 fs are evident. The dynamics of the differential transmission ∆*T* at a fixed position (blue circle) in the transparent region close to the antenna gap is shown in Fig. 3b. Interestingly, near the antenna rim, the electron transmission becomes larger than in the absence of the pump, in contrast to the signal drop created by the released electron cloud. This signal increase persists for delays beyond our measurement range, as is shown exemplarily in Fig. 3b (red circles).

**Fig. 3:**
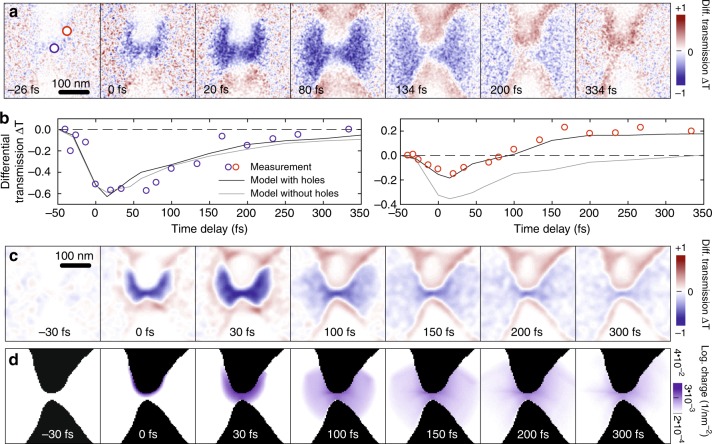
**Differential UPEM transmission images and electron trajectory simulations.** **a** Series of differential UPEM images created by subtracting a background image recorded at *τ* = −66 fs from the transient UPEM images similar to those in Fig. 2a, but recorded for a pump field strength of 0.7 V/nm. A transient reduction in electron transmission is color-coded in blue and is dominant in the gap region. A transient increase in transmission (red) appears at later delay times in the two vertical arms of the antenna. Here, electrons are deflected into a non-transparent region due to residual positive charges on the antenna arms. The charging is more pronounced in the upper antenna arm acting as the electron source. **b** Temporal evolution of the differential transmission at the two positions marked in a (open circles), together with the simulated evolution derived from a model without (gray curves) and with (black curves) holes. **c** Simulated differential UPEM images revealing both the expanding shadow in the gap region and the transmission enhancement in the antenna arms. **d** Transient evolution of the charge density deduced from the trajectory simulations, plotted on a logarithmic color scale

We have performed classical electron trajectory simulations to understand these dynamics. In these simulations, we model the probe electrons as a beam of single point-like charges that are deflected by the Coulomb fields generated by a randomly distributed cloud of photoelectrons. These electrons are placed at the surface of the nanoantenna in a 50-nm-wide region matching the local surface plasmon mode profile of the gap antenna. The electrons are created within a time window set by the pump laser, and the kinetic energies are randomly chosen. For simplicity, we assumed a uniform distribution of their velocities up to a maximum set by the photon energy. Details on these simulations can be found in the Methods section and in the Supplementary Information. Simulations of the resulting differential transmission images are shown in Fig. 3c. For each image, 300,000 simulation runs were performed. For each run, the photoelectron distribution in the nanogap region was calculated from randomly chosen starting conditions and sensed by one probe electron. When the appropriate number of released electrons is selected, 30 per pulse, the space-time dynamics of the experimentally observed drop in differential transmission is accurately reproduced. This strongly supports the notion that the deflection of the probe electrons quantitatively maps the expansion of the photoreleased electron cloud, a concept further substantiated by earlier works that have studied similar phenomena, albeit with lower spatio-temporal resolution^29,43–45^.The space-time dynamics of the electrons released from the antenna rim that is predicted by our model simulations is shown in Fig. 3d. Evidently, these simulations well account for the differential transmission dynamics in the transparent region of the nanoantenna. However, we cannot reproduce the persistent increase in differential transmission that is observed in Fig. 3a for probe positions near the antenna rim if we restrict the simulations only to a light-driven release of photoelectrons. Instead, we are led to assume that photoemission results in a build-up of positive charges at the metal surface near the apices of the two antenna arms. We have added an appropriate number of positive charges on each of the arms in our simulations. As is evident from Fig. 3c, this leads to a deflection of probe electrons into the otherwise obscured, non-transparent regions in the outer rim of the antenna arms. Directly at the edge of the antenna, the finite spatial resolution of our electron probe leads, initially, to a decrease in transmission owing to the release of photoelectrons, whereas, at later times, the transmission enhancement owing to the positive charge deflection dominates. Representative ∆*T* dynamics are shown in Fig. 3b (red circles). This transition between negative and positive ∆*T* can only be understood by assuming that the photoemission results in a persistent positive charging of the upper arm of the metal antenna. In principle, the observed charging may be accounted for either by photoinduced holes at the inside of the metal or by positively charged, long-lived surface states. Conceptually similar studies of photoelectron deflection by charge-separated electric fields have been previously performed with picosecond temporal resolutions and tens of microns spatial resolution, e.g., on cluster plasmas^44,46^, copper film surfaces or near graphite surfaces^29^. Our UPEM technique advances the space-time resolution of such deflection techniques to the 10 nm/10 fs regime, opening up exciting avenues for probing photoinduced charge transfer and separation dynamics in individual nanostructures^37^. At present, the time resolution is still slightly too low to resolve the intrinsic plasmon dynamics in small nanostructures but sufficient for probing the effects of electron-phonon interactions on these dynamics^3^.

This substantial improvement in space-time resolution has been accomplished by implementing plasmonically enhanced multi-photon photoemission from sharp metal tapers, creating a free-standing source of ultrafast photoelectron pulses. In a proof-of-principle experiment, we have used this source here to study the ultrafast release and expansion of a cloud of charges from a single nanometer-sized plasmonic antenna, providing a direct visualization of electron dynamics and charge separation in nanostructures at ultrafast time scales.

At present, our UPEM uses low kinetic energy electrons to probe charge carrier dynamics with 20-nm spatial and 25-fs temporal resolution. The comparatively low velocity of the electrons increases the interaction time with local electric fields in the vicinity of the nanostructure and thus enhances angular deflections of the passing electrons. This makes low-energy UPEM extremely sensitive to weak quasistatic electric or transient optical fields at the surface of small nanostructures. For the current spatial resolution of 20 nm, we estimate that local electric fields with amplitudes as low as 2 × 10^7^ V/m may still be sensed by the passing probe electrons (see the Supporting Information). The low kinetic energy of the probe electrons necessarily limits transmission microscopy to ultrathin, monolayered samples, whereas fields at the surface of larger nanostructures can be probed for free-standing samples or in reflection geometry. For free-standing samples, such as those studied here, the time resolution is ultimately limited by the transient time of the probe electrons across the optical near-field, ~ 7 fs or 1.2 optical cycles in our experiments. Using smaller nanostructures and/or faster electrons, the probe electrons can transit the electromagnetic near fields close to the nanostructure surface in less than half an optical cycle, which allows for direct probing of the inherent dynamics of optical near fields of single nanostructures. Under such idealized half-cycle conditions, the sensitivity to transient optical fields is as high as that to quasistatic fields. For longer transit times or electron pulse durations, the electrons undergo a quiver motion, and the sensitivity is reduced accordingly. Furthermore, when investigating sufficiently small nanostructures, the resulting UPEM images show pronounced interferences between incident and scattered electrons. Hence, one can make use of existing in-line holography schemes to improve the spatial resolution to below 1 nm^42^ and to expand these schemes from static imaging towards dynamic holography and to the recording of ultrafast dynamics on single nanostructures. Because the phase of the electron wave after interaction with the nanostructure is encoded in the recorded hologram, quantitative information about the rapidly changing local electric fields is accessible. This promises imaging of coherent electrodynamic fields near surfaces with nanometer spatial and sub-cycle temporal resolution and may be key for probing local and ultrafast charge carrier dynamics in nanostructures by deflecting passing electrons^11^.

## Materials and methods

### Tip and sample preparation

Single-crystalline gold nanotips were fabricated from polycrystalline gold wires with a diameter of 125 µm. After cleaning in ethanol, the wires were annealed at 800 °C for 8 h and then slowly cooled over another 8 h to room temperature. These annealed wires were then electrochemically etched in HCl (aq. 37%). After inspection by scanning electron microscopy, tips with a diameter of < 20 nm and with grain boundaries suitable for SPP coupling were selected.

The plasmonic nanoresonator shown in Fig. 1d was prepared in a free-standing gold film with a thickness of 30 nm. The free-standing Au film was prepared using a commercial TEM window grid with 10-nm-thick silicon nitride membranes. A 30-nm-thick Au film was sputtered onto the top side of the windows, and subsequently, the 10-nm silicon nitride membrane was removed by reactive ion etching in CF_4_ plasma. Two circular rings with a radius of 200 nm separated by a center-to-center distance of 450 nm were milled into the gold film with a focused gallium ion beam microscope, and the rings were connected by a 30-nm-wide channel. More information on tip and sample preparation can be found in the supporting online material.

### Simulation of UPEM images

The simulated UPEM images presented in Fig. 3 were obtained by calculating the classical trajectories of individual probe electrons deflected by the Coulomb interaction with a randomly distributed cloud of photoelectrons released from the plasmonic nanoresonator. At the start of each simulation, 30 electrons are generated along a curve along the rim of the upper antenna arm in the sample plane. The randomly chosen emission spots follow a Gaussian distribution with a full width at half maximum (FWHM) of ~ 50 nm, centered in the middle of the antenna arm. The initial kinetic energy of the electrons is uniformly distributed between 0 and the photon energy of 0.7 eV, and their emission time is randomly chosen from a Gaussian distribution with a FWHM of 15 fs. The motion of the emitted electrons is restricted to a 2D plane in the center of the nanoresonator, and the direction of their initial velocity is randomly distributed. The electrons that collide with the metal surface are either absorbed (with 10% probability) or elastically reflected.

Probe electrons with a kinetic energy of 60 eV are released from the tip apex (a point source placed along the detector-sample axis at a distance of 2700 nm from the antenna center). The birth time of each probe electron is randomly chosen from a Gaussian distribution with a FWHM of 20 fs. The electrons arrive in the sample plane at time delay _*τ*_ with respect to the emission of the electrons released from the nanoresonator. Probe electrons that hit the metal surface of the nanoresonator during their trajectory are fully absorbed. The propagation and interaction of all 31 electrons, i.e., a single probe electron and 30 photoemitted electrons, is calculated by solving Newton’s equation using the classical Runge-Kutta method with a step size of 1 fs. The simulation continues for a simulation time of 75 fs until the probe electron is well outside the interaction region. The impact position of the probe electron on the detector screen is calculated from its terminal propagation angle at the end of the simulation. At each time delay τ, the results from 300,000 simulation runs are added to generate a two-dimensional map of the electron impact positions on the detector. These point-projection images are then calculated for a series of time delays *τ*. We have tested that the restriction of the motion of the electrons emitted from the nanoresonator to a two-dimensional plane through the center of the resonator does not significantly affect the resulting point-projection images. A comparison of the results from a 2D simulation with those from a fully three-dimensional (3D) model in which the photoemitted electrons from the nanoresonator are initially distributed in a 3D volume with a thickness of 30 nm is shown in the Supporting Information.

## Electronic supplementary material

Supplementary Information

Supplementary Movie 1

Supplementary Movie 2
