# Supplementary material for: Observing charge separation in nanoantennas via ultrafast point-projection electron microscopy
Source: Light Sci Appl. 2018 Aug 22;7:55. doi: 10.1038/s41377-018-0054-5 (PMC6107015; doi:10.1038/s41377-018-0054-5)
Supplement: Supplementary file 1 — Supplementary Information [file 41377_2018_54_MOESM1_ESM.pdf]

Supplementary Information
for

Observing charge separation in nanoantennas via ultrafast point-projection electron microscopy

Jan Vogelsang, Germann Hergert, Dong Wang, Petra Groß, Christoph Lienau

e-mail: jan.vogelsang@uni-oldenburg.de,
christoph.lienau@uni-oldenburg.de

1. Gold nanotips

Single-crystalline gold nanotips were fabricated from polycrystalline gold wires (99.99%) with a diameter of 125 µm (commercially available from Advent Research Materials), as described before[1]. After cleaning in ethanol, the wires were annealed at 800 °C for 8 h and then slowly cooled over another 8 h to room temperature. These annealed wires were then electrochemically etched in HCl (aq. 37%). For etching, rectangular voltage pulses with a frequency of 3 kHz and a duty cycle of 10% were applied between the wire and a platinum ring serving as the counter electrode. The tips were inspected by scanning electron microscopy and tips with a diameter of less than 20 nm were selected. All tips show grain boundaries at the interface between differently oriented facets. We selected tips with grain boundaries that are suitable for SPP coupling.

2. Plasmonic nanoresonators

The plasmonic nanoresonator shown in Fig. 1d in the main manuscript has been prepared in a free-standing gold film with a thickness of 30 nm (see Supp. Fig. 1). The free-standing Au film was prepared using a commercial TEM window grid with 10-nm thick silicon nitride membranes (Plano GmbH, window size: 100×100 µm). The 30-nm thick Au film was sputtered onto the top side of the windows, and subsequently the 10-nm silicon nitride membrane was removed by reactive ion etching in CF_4 plasma (Oxford RIE 100). A dual beam focused Gallium ion beam microscope (FEI Helios 600i), operated at a beam current of 1.1 pA, has been employed for milling two circular rings with a radius of 200 nm. The rings were separated by a center-to-center distance of 450 nm and connected by a 30-nm-wide channel.

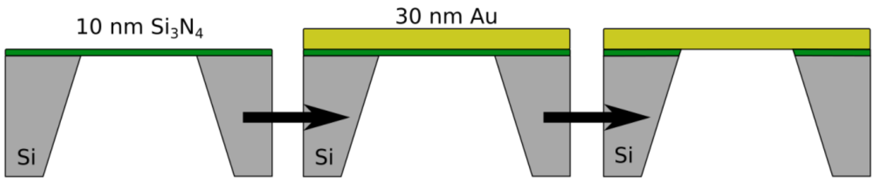

Supplementary Figure 1 | Schematic illustration of the fabrication of a freestanding 30-nm thick gold film. A 30-nm thick gold film is sputtered onto a 10-nm thick silicon nitride membrane. The membrane is subsequently removed by reactive ion etching.

3. Experimental setup

A schematic of our experimental setup is shown in Fig. 1a of the main manuscript. In this setup, few-cycle femtosecond pulses at a wavelength of 1.8 µm and with a duration of 15 fs are supplied at a repetition rate of 5 kHz by a home-built noncollinear optical parametric amplifier system[2]. The pulses are split into two beam paths using a thin 50/50-beamsplitter with low group delay dispersion. The pulses in the first path are attenuated with a reflective neutral density grey filter to a pulse energy of 0.9 nJ and are directed towards the gold taper for electron emission. The 18-mm-diameter beam is focused with a parabolic mirror through a

1.5-mm thick CaF_2 window of an ultrahigh vacuum chamber that contains the home-built ultrafast point-projection electron microscope (UPEM). The vacuum chamber is evacuated to a base pressure of $5\cdot10^{-10}$ mbar using a combination of a turbo molecular and an ion getter pump.

The laser beam is focused to a spot with a radius of 4.7 µm and the focal spot is aligned on a grain boundary at the shaft of our conical gold tapers. The selected grain boundary has a distance of 80 µm from the taper apex and acts as a single-slit grating coupler. It couples the incident light to surface plasmon polaritons (SPPs) propagating along the taper shaft. SPP nanofocusing[3-5] results in a locally enhanced SPP field, which induces electron emission from the taper apex. The photoemitted electrons serve as probe electrons in our time-resolved UPEM.

The pump pulses in the second beam path are attenuated to ~3 nJ and are focused onto the sample, using identical optical elements. A flat mirror is used to steer the beam such that the plasmonic nanoresonator sample is illuminated on the side that is opposing the metal tip and facing a microchannel plate electron detector. The pulses are incident under an angle of 58° to the surface normal and are focused to a ~9-µm-radius spot on the nanoresonator surface. The arrival time of the pulses at the sample is adjusted by a combination of a manual translation stage and a piezo-actuated stage with a travel range of 100 µm for fine adjustment.

The sample is placed at a distance of 2.7 µm from the taper apex and oriented such that the long axis of the gold taper is perpendicular to the sample plane. The distance between taper apex and sample is controlled by a slip-stick stage with a travel range of 20 mm and an accuracy of 1 nm (Attocube ECS3030). The section that is imaged can be selected by laterally shifting the sample using two slip-stick stages that are identical to the first.

The probe electrons are recorded with a detector that is placed 75 mm behind the sample. It consists of a microchannel plate (MCP) of 45-mm diameter, followed by a 40-mm diameter P43 phosphor screen. The emission pattern is recorded by a CCD camera (PCO Pixelfly USB) with 1392 x 1040 pixels.

In the experiments, we have applied a DC bias voltage of -20 V to the tip. The sample bias is set at +40 V and the detector is grounded. This results in an average kinetic energy of the probe electrons in the sample plane of 60 eV, sufficient to overcome the repulsive potential between sample and detector. Low kinetic energy electrons emitted from the nanoresonator by the pump pulse (sample electrons) are blocked by the 40 V potential difference between sample and detector.

Time-resolved UPEM images have been recorded by arbitrarily selecting pump-probe time delays covering an interval of ~500 fs. For each delay position, electrons are accumulated over a total of 7 minutes by acquiring a series of camera images with an integration time of 400 ms each (2000 laser pulses). A peak-finding routine determines the electron impact positions on-line after acquisition of each image. Typically, about 1000 electrons are found per image, and their impact positions are stored. Ultrafast transmission images for a given time delay Δt are constructed from all $1.05\cdot10^{6}$ stored impact positions. For this, the transmission image $U\left(x,y,\Delta t\right)$ is taken as the number of electrons $N\left(x,y\right)$ that is recorded at the impact position $\left(x,y\right)$ at time delay Δt. Due to the negligible probability of electron

transmission through the metallic part of the nanoresonator these impact positions provide a clear map of the geometric shape of the nanoresonator in the region outside the expanding electron cloud (Figs. 1e, f). In an off-line analysis, we therefore used these maps to track a minor drift of the sample by comparing the sample position for different time delays. The electron impact positions are corrected accordingly. A series of these images recorded at a number of different time delays results in a movie, showing the deflection of the probe electrons by the ultrafast charge cloud in the nanoresonator.

Two of these movies, recorded at slightly different pump intensities, are shown as Supplementary Movies 1 and 2. All images in these movies and also the images shown in the main manuscript are normalized images that have been obtained by normalization of a background transmission signal. This background signal is given by the transmission in the fully transparent part of the plasmonic structure, well outside the nanoresonator. It has been obtained by fitting a 2nd order two-dimensional polynomial to the transmission signal at 11 sample points on the very left and very right of the transparent region.

4. Plasmon-assisted electron emission from metallic nanotips

As discussed in Sec. 3, surface plasmon polariton (SPP) wavepackets have been launched at the surface of the gold nanotip by coupling 15-fs light pulses, centered at 1.8 µm, to a grain boundary on the gold nanotaper separated by 80 µm from the apex. SPP propagation and nanofocusing along the shaft result in the formation of a nano-localized surface plasmon hot spot at the very apex of the taper[3-5]. We estimate a pulse energy of the localized spot of about 200 fJ, which corresponds to a maximum electric field strength at the taper apex of around 5 V nm^{-1}.[6] This is sufficiently high to induce multiphoton photoemission of electrons. From the ratio of the work function (5.5 eV for gold) and the photon energy, we expect that the photoemission is induced by a seven photon ($N = 7$) nonlinear process. Experimentally we typically find somewhat lower nonlinearities of $N = 5$ to $N = 6$, most likely due to the slight DC bias voltage of about 60 V that is applied to the tip. This bends the vacuum potential in the near field region around the tip apex and locally reduces the effective work function. Also, the transient heating of the electron gas by the ultrafast excitation pulse may contribute to the reduction in effective nonlinearity[4].

Under these excitation conditions we detect around 2500 electrons per second, which, taking into account the detection efficiency, corresponds to about one electron being emitted per laser pulse. To test the spatial confinement of the localized electron source, we have used the source for recording electron diffraction patterns from single carbon nanotubes[7]. From the resulting interference fringes we deduce a radius of the emitter area of less than 5 nm.

To determine the temporal duration of the localized SPP (LSP) pulse at the taper apex, we perform a cross correlation measurement between the LSP and a second laser pulse. For this, we remove the sample from the beam path and focus the second laser beam, which normally excites the sample, onto the apex. The focused laser field and the LSP field at the apex interfere to induce multiphoton photoemission. To ensure that the two fields are of approximately equal electric field strength we adjust the laser pulse energy of each optical path independently. When only one of the fields is present at the apex, as few as 10 electrons are emitted during the detector integration time of 400 ms. In contrast, if both fields are present and the delay is adjusted to maximum pulse overlap, the electron count rate increases

to 2500 electrons in 400 ms. When changing the time delay of the second laser pulse with respect to the LSP, the field interference is periodically modulated, resulting in a nonlinear cross correlation measurement. Figure S2 shows a measured cross correlation as blue circles, together with a calculated cross correlation as the red curve.

From the width of the central interference fringe, a nonlinearity of the emission process of 5 is deduced. This is lower than the number of photons needed to overcome the work function of 5.5 eV of gold since the DC field applied between the tip and the detector bends the local vacuum potential near the taper apex and this effectively reduces the work function to ~ $0.7\,\mathrm{eV}\cdot 5 = 3.5\,\mathrm{eV}$ [8-10]. Also, the transient heating of the electron gas due the short laser pulse excitation may contribute. By fitting the measured data (red curve in Supp. Fig. 2) with a cross correlation of simulated laser and plasmon fields, we deduce a duration of the LSP pulse of 18 fs (full width at half maximum of the pulse intensity). Considering the nonlinearity of the electron emission process of $N \approx 5$, this indicates that the electrons are emitted from the apex during a much shorter time window of $18\,\mathrm{fs}/\sqrt{5} = 8\,\mathrm{fs}$.

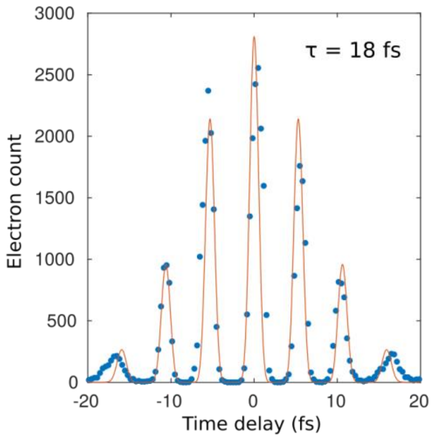

Supplementary Figure 2 | Temporal duration of the localized plasmon (LSP) pulse at the apex of the gold nanotaper. The temporal duration of the LSP after plasmonic nanofocusing is measured by a nonlinear cross correlation of the LSP and an external laser pulse with 15 fs duration. The electron emission process with a nonlinearity of 5 is used to deduce a localized plasmon duration of 18 fs at the taper apex.

The small opening angle of the taper leads to a strong increase of the static electric bias field in the region around the tip apex. For sharp tips, as used in the present experiments, these fields decay on a length scale of a few nm[9]. Since the photoemitted electrons are accelerated by this field, the electrons gain the major part of their kinetic energy already within the first tens of nanometers distance from the taper apex. The final kinetic energy, which they have in the sample plane 2.7 µm away from the taper apex, is largely given by the applied DC bias and amounts to 60 eV. Assuming a width of the kinetic energy distribution of the electrons of 2.5 eV, which is typical for multiphoton photoemission, we calculate an electron pulse duration of 20 fs (FWHM) in the sample plane. This compares well to the experimentally demonstrated upper limit for the temporal resolution of 25 fs (see Fig 2d in the main text).

5. Photoemission from the plasmonic nanoresonator

Electron emission was induced in the gap region by illuminating the nanohole resonator with short laser pulses with a peak electric field strength of 0.6-0.7 eV. Without probing electrons, $N_{\text{det}} = 3$ electrons per laser shot were recorded on the MCP detector when setting the DC bias potential of the tip to -20 V and that of the sample to -5 V, while keeping this MCP at ground. Under these conditions, the low kinetic energy electrons that are photoemitted from the plasmonic resonator are no longer blocked from the MCP detector. We estimate the total number of electrons emitted from the nanoresonator (sample electrons) by photoemission as follows: We consider that the emitted electrons are accelerated in the direction of the detector in a large solid angle of about 1.0 sr. The detector only covers a solid angle of 0.2 sr, such that the number of detected electrons is reduced by a factor $F_1 = 1/5$. The detection efficiency of the MCP is about 50%, giving a second factor $F_2 = 1/2$. In total, the number of electrons that are photoreleased from the nanoresonator per laser shot is estimated to be $N \approx N_{\text{det}} \cdot (F_1 \cdot F_2)^{-1} = 30$.

6. Model for the temporal transmission change induced by photoemission from the plasmonic nanoresonator

Fig. 2d of the main text shows how the transmission of probe electrons through the sample plane is affected by release of photoelectrons from the gap of the plasmonic nanoresonator. To model the measured transmission data in Fig. 2d, we used a time-delayed response of the system that is convolved with a Gaussian distribution which accounts for the finite time resolution of the experiment. The following expression $F(t)$ is taken to describe the time-delayed response:

$$F(t) = 1 - A \cdot \Theta(t - t_0) \cdot e^{(t-t_0)/t_1} \tag{1}$$

Here, A is the amplitude of the transmission drop, and $\Theta(t - t_0)$ is the Heaviside function. This assumes that electrons that are photoreleased from the resonator gap at time zero will block the probe electron transmission only after a certain time delay t_0. This time delay varies with the distance between gap center and probe position and defines the time that it takes for the fastest released electrons to propagate from the gap to the probe position. In accordance with experimental observations, we assume an exponential recovery of the transmission with a time constant t_1. This time constant essentially reflects a typical time scale for the spreading of the photoreleased electron cloud. In Fig. 2d, we find that t_1 varies slightly from 185 fs for the curve on the left to 150 fs for that on the right. To simulate the experimental data, we calculate the convolution integral between the response function in Eq. (1) and a Gaussian distribution $G(t) = \exp\left(-4\ln(2)t^2 / \tau_E^2\right)$. For a probing position close to the gap, the width of the Gaussian τ_E reflects mainly the temporal duration of the probing electron beam (FWHM of 25 fs for the curve on the left). For larger distances, the width increases slightly (to 35 fs

for the curve shown on the right), which is likely to be caused by an additional slight acceleration of the fastest electrons due to repulsion by the slower cloud electrons.

7. Numerical Simulation of UPEM images

The theoretical modelling of stationary point-projection electron microscopy images is highly developed[11-13]. Essentially the tip emits a coherent, quasi-monochromatic electron wave of predominantly spherical symmetry. This electron wave is diffracted off the object and interferes with the transmitted incident wave. The resulting in-line hologram is recorded on the detector screen. The shape of the object can then be reconstructed from this hologram by deconvolution. In the present experiments, the de Broglie wavelength of the probe electrons with 60 eV energy in the sample plane is 0.15 nm and thus much smaller than the typical dimensions of the plasmonic nanoresonator (thickness ~ 30 nm, edge sharpness ~ 10 nm). Hence the experimental data can reasonably well be described in the ray tracing limit, treating the electrons as classical particles with sub-relativistic velocities. In this limit, the interaction of the probe electrons with the charges that are photoreleased from the antenna mainly results in a slight deflection of the probe electron trajectory by an angle φ. Estimates of the Coulomb forces between probe and photoreleased electrons and the resulting deflections are given in Sec. 9.

To simulate the transient UPEM images we have therefore calculated the trajectories $\vec{r}(t)$ by solving Newton's equation of motion

$$m_e \frac{d^2\vec{r}(t)}{dt^2} = \sum_i \frac{-e \cdot q_i}{\left|\vec{r}(t) - \vec{r}_i(t)\right|^3}\left(\vec{r}(t) - \vec{r}_i(t)\right) \tag{2}$$

for a single probe electron experiencing the Coulomb forces that are exerted by all charges that are photogenerated from the nanoresonator on the probe electron.

The trajectories of the probing electron and all electrons that are released from the sample are calculated using the classical Runge-Kutta method with a fixed step size of 1 fs. For each simulation run, 30 photoreleased electrons are modeled within the sample plane, and a single probing electron propagates through this plane. Time zero is defined as the time when electrons are photoreleased from the sample, and the delay time of a certain simulation run is given by the arrival time of the probe electron in the sample plane.

The probing electron originates spatially from a point source along the detector-sample axis and at a distance of 2700 nm from the sample. The arrival time of each probing electron in the sample plane is randomly chosen from a Gaussian distribution with a FHWM of 20 fs to model the finite temporal duration of the electron pulse in the sample plane. The initial kinetic energy is set to 60 eV. Its propagation direction is randomly chosen, such that the probe electrons equally cover the sample area of interest (diameter 300 nm).

For the simulations, the three-dimensional shape of the nanostructure was deduced from the SEM image shown in Fig. 1c and the known 30-nm thickness of the gold film. Since the transmission of probe electrons through the metallic part of the antenna is completely negligible, we assume that all probe electron trajectories that hit the metal film are fully

absorbed. The simulated images are then filtered with a two-dimensional Gaussian kernel to mimic the finite spatial resolution in the experiment. In this way, simulations performed without photoemission from the nanoantenna closely reproduce the transmission image that is shown in Fig. 2b of the main manuscript. For collisions between the slow sample electrons and the metal surface, we assume, in contrast, that the electrons are re-absorbed with 10% probability and otherwise the collisions are treated as elastic reflections.

To simulate the effect of photoemission on the probe electron trajectories, we first estimate the spatial near-field distribution in the region around the upper antenna arm. Photoemission is restricted to this arm, since the images in Fig. 2a show that illumination by the pump laser predominantly releases electrons from this arm. This asymmetry is assigned to slight shape differences between both arms resulting in a higher field enhancement in the upper arm. The near-field distribution defines the spatial origin of the electrons that are photoemitted from the nanoresonator by the ultrafast pump laser. Due to the highly nonlinear emission process, photoemission is restricted to a narrow region with a Gaussian distribution of 50 nm FWHM around the apex of the upper antenna arm. In the simulations, thirty sample electrons are created at randomly chosen positions within this localized near field. The spatial distribution of the released electrons can be seen in Fig. 3d of the main manuscript (for zero time delay). They propagate away from the upper nanoantenna arm with velocities that are equally distributed between zero and the maximum velocity, which is set by the laser photon energy of 0.7 eV. To account for the finite time resolution of the pump pulse, the birth time of the sample electrons is randomly chosen in time with a probability distribution given by the pulse duration of the pump laser of 15 fs.

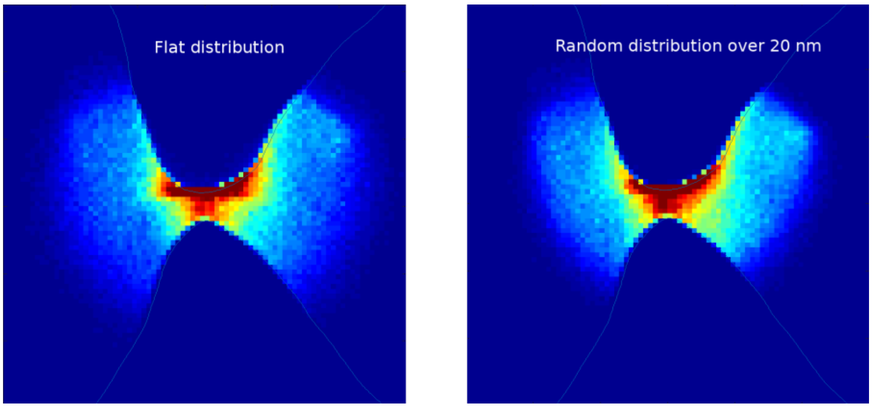

Supplementary Figure 3 | Electron density distributions. Projected electron density at t = 100 fs for different initial distributions of the electrons released from the nanogap: Left, for a 2D simulation, and right, for a fully 3D simulation in which the electrons are distributed over a 20-nm thick volume inside the gap resonator.

For the simulation, the motion of the electrons in the cloud was restricted to a 2D plane. To confirm that this is sufficient to model the cloud expansion, we initially performed fully three-dimensional simulations of the electron cloud and compared them to the results of the 2D model (see Supp. Fig. 3). We found that the charge cloud expansion can similarly well be modelled in a computationally simpler two-dimensional charge expansion model. This can be directly seen from the representative comparison of the electron densities shown in Supp.

Fig. 3. Consequently, we distribute the electrons of the charge cloud in a two-dimensional plane in the following calculations.

By comparing the simulations to the experiment, we found that a good match with the experimental observations could only be achieved if it is assumed that the photoemission of the sample electrons results in a persistent, positive charging of the upper and lower antenna arm. To account for this, we have assumed that the photoemission of the sample electrons results in a positive charging of the upper arm. We consider this effect by placing, at the moment of photoemission, a positive charge of +19 e in the center of the upper antenna arm, at a distance of 120 nm from the center of the antenna gap. In addition, we assume that the photoreleased electron cloud gives rise to the creation of an image charge in the lower antenna arm. We model this by placing a charge of +4 e in the center of the lower antenna arm, at a distance of 70 nm from the center of the antenna gap. The positive charging of the sample is always kept in proportion to the number of free sample electrons, i.e., the absorption of sample electrons by the metal is assumed to reduce the net positive charge on the metal antenna accordingly. Thus, a fraction of the positive charging remains until the end of the simulation. The spatial position of the two positive charges is kept fixed during the simulation.

Simulations are performed for a series of delay times τ between the pump pulse, ejecting electrons from the nanoresonator, and the arrival time of the electron probe pulse in the sample plane. The simulation starts at $\tau = -50\ \text{fs}$. At each time step, the forces between all charges are calculated and the trajectories of all electrons are simulated according to the acting forces. For each probe electron, the terminal propagation direction is calculated 75 fs after the probing electron has passed the sample plane. This propagation direction is used to calculate its impact position on the electron detector at a distance of 75 mm. For of each image shown in Fig. 3c in the main text, a total of 300.000 simulation runs are performed and summed up.

8. Effects of photoemission on UPEM images

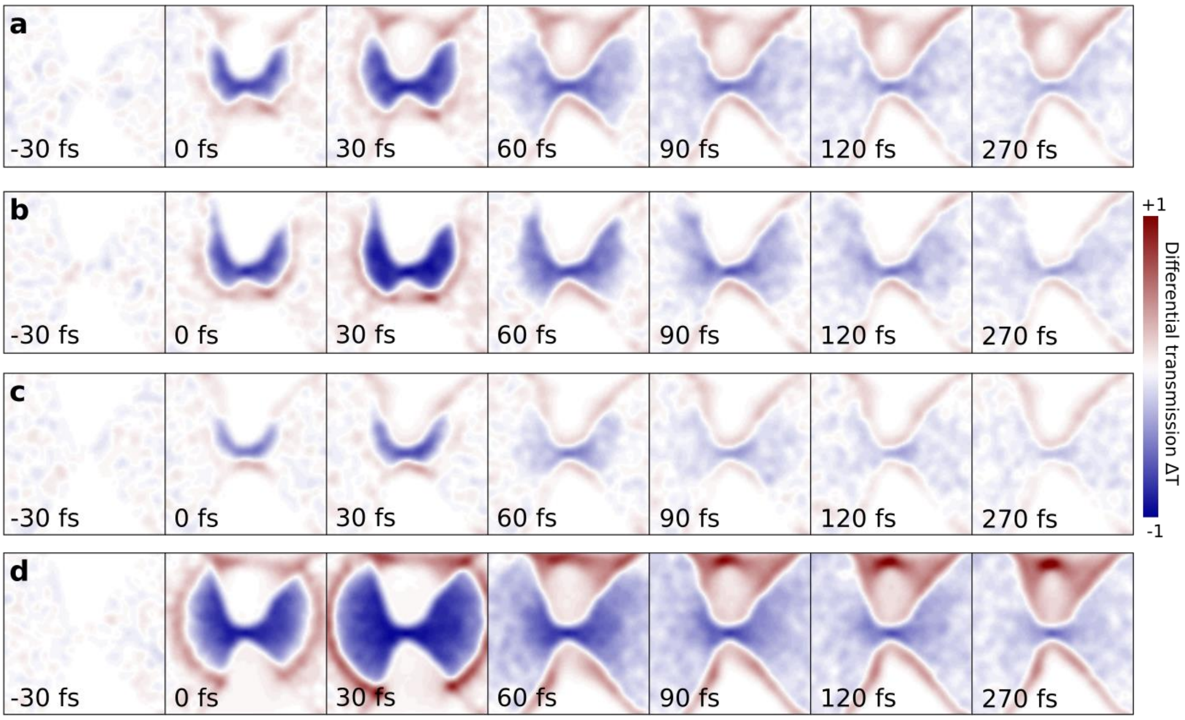

Supplementary Figure 4 | Simulated differential transmission images. a, Simulated UPEM differential transmission images for different time delays of the electron probe with respect to the optical excitation of the plasmonic nanoresonator. The electron trajectories were calculated for a distribution of 30 electrons released from the sample, and assume a positive charging of the sample (same as Fig. 3c of the main text). **b**, The same simulations as in **a**, but neglecting the positive charging of the sample. **c**, A similar series of differential transmission images calculated under the assumption that only 10 electrons are photoreleased from the sample, and **d**, for 100 photoreleased electrons. In **c** and **d**, the ratio of positive charges in the sample to the released negative charge was the same as in **a**.

We have performed simulations of electron trajectories and of the resulting differential transmission images as described in Sec. 7 for a series of different photoemission scenarios. In these simulations, we have systematically varied the underlying assumptions about the pump-induced photoemission from the plasmonic nanoresonator. Some representative results are summarized in Supp. Fig. 4.

The first simulated time series (Supp. Fig. 4a) is the same simulation as shown in Fig. 3c of the main text. These are the simulations in which we have achieved the most convincing agreement with the measurement (Fig. 3a of the main text). For this, we have assumed that a total of 30 photoelectrons are released from the upper arm of the plasmonic nanoantenna by the pump laser. In addition, we considered that the photoemission results in positive charging of the upper arm and that image charges are induced in the lower arm. The magnitudes of the charges are dynamically adapted, as described in Sec. 7.

Most of the free parameters in this simulation are readily obtained from the experiment. (i) The differential transmission images in Fig. 3a recorded around time zero show directly that

the pump laser photoemits electrons from a narrow region around the apex of the nanoresonator. This is the region of maximum field enhancement near the gap. (ii) The maximum kinetic energy of the released electrons defines the spreading velocity of the photoreleased electron cloud and, thus, the variation of the time shift t_0 in Eq. (2) with probe position. A maximum kinetic energy of 0.7 eV matches well the data in Fig. 2d and matches the photon energy of the driving laser in our experiment. (iii) A broad distribution of kinetic energies between 0 eV and 0.7 eV is needed to mimic the slow exponential recovery of transmission with time constant t_1. This essentially leaves the number of photoreleased electrons and photoinduced generation of positive charges on the antenna arms as the open parameters in the simulations.

To address the second point, we have repeated the simulation shown in Supp. Fig. 4a, again with 30 photoreleased electrons per laser shot, but without positive charges remaining inside the sample (Supp. Fig. 4b). Neglecting the positive charging does not perceivably alter the expansion of the photoreleased electron cloud. The experimentally observed drop in transmission is similarly well reproduced as in the first example. The pronounced increase in differential transmission for probe positions near the antenna rim is, however, not reproduced. Experimentally, we see that probe electrons that pass the nanoresonator gap close to the antenna rim are deflected into the region that is obscured by the metal if no pump pulses are present.

In simulations without positive charging, we observe a faint increase in differential transmission signal within a rather narrow range inside the antenna arm. This area, however, is much smaller and the increase is much less pronounced than in the experiment. This is particularly apparent in the upper arm. This faint increase decays with the same time constant t_1 as the main transmission dip inside the antenna gap. This positive signal at the rim of the antenna is a result of the repulsion of probe electrons by the sample electrons, which push probe electrons into the otherwise obscured area inside the antenna rim.

In contrast, the experimentally measured signal is of larger amplitude, appears in a much broader region (Supp. Fig. 4a) and shows a distinctly different time dynamics, persisting much longer than t_1. We found that we can only reproduce this experimental feature by assuming a long-living positive charging of the antenna. This positive charging deflects probe electrons into the region that is otherwise obscured by the metal and results in the persistent positive differential transmission signal that is clearly seen in Fig. 3b and Supp. Fig. 4a (right) for time delays of more than 150 fs. Somewhat less pronounced positive differential transmission signals are also visible in the lower antenna arm (Fig. 3a). Also, these signals are better reproduced by assuming a persistent positive charging of the lower antenna arm, e.g., due to the formation of image charges by the spreading electron cloud. We therefore concluded that we needed to include a positive charging scenario to obtain an acceptable match between simulation and experiment. This positive charging is also included in the simulations that are shown in Supp. Figs. 4c and d.

Having found an acceptable modelling of the positive charging effects, this now leaves the total number of pump-released electrons as essentially the only free parameter in the model. In Supp. Figs. 4c and d we show simulation results for a much lower number of 10 (c) and a

much higher number of 100 released electrons (d). In both simulations, we kept the ratio of positive to negative released charges the same as in Supp. Fig. 4a.

It is evident that the differential transmission signals that are predicted for 10 electrons are much smaller than those found experimentally.

In contrast, when increasing the number of photoreleased charges to 100, the amplitude of the differential UPEM signal is clearly much larger than that observed experimentally. A convincing agreement has been found for 30 photoreleased electrons, which is in excellent agreement with the number of photoreleased electrons by the pump that is estimated from the independent measurement described in Sec. 5.

9. Forces acting during electron deflection

We have seen in the previous section, that the reported UPEM experiments can reasonably well be described in a classical ray tracing limit in which the Coulomb forces that are induced by the pump-released charge in and around the antenna lead to a deflection of the probe electron trajectory. In the ray tracing limit the deflection angle φ of the probe electrons that have passed through the sample plane is given as

$$\tan\varphi = \frac{\Delta \mathrm{v}_{\|}}{\mathrm{v}_z} \qquad (2)$$

Here the axial velocity $\mathrm{v}_z \approx 4.5$ nm fs^{-1} of the probe electrons is given by their initial kinetic energy (60 eV). Hence, the probe electrons need about $\Delta t =$ 6.7 fs to pass across the 30-nm thick metal film. The interaction-induced change in lateral, in-plane velocity $\Delta v_{\|}$ can be determined from the measured deflection angle. We experimentally find maximum deflection angles of $\varphi = 0.05\,\mathrm{rad}$, corresponding to an in-plane velocity of $\Delta v_{\|} = 0.22$ nm fs^{-1}. Such a deflection is induced by a time-averaged in-plane force $\langle F_{\|} \rangle = m_e \Delta v_{\|} / \Delta t \approx 30$ pN. Such a force is exerted by two elementary charges at a distance of ~3 nm, which roughly agrees with the average distance between the 30 electrons directly after emission from the $\sim (10\,\mathrm{nm})^3$ volume in the gap region.

The minimum detectable deflection is a measure for the sensitivity of our UPEM. The spatial resolution of 20 nm demonstrated in Fig. 2c corresponds to a displacement on the detector screen of 0.8 mm or an angular resolution of 0.01 rad. Such a small deflection would be caused by a force of 2.7 pN acting during the interaction time of 10 fs, or an electric field strength of 0.02 V/nm.

Supplementary Movie 1 | Ultrafast point-projection electron movie #1

A plasmonic nanoantenna is illuminated with ultrafast laser pulses with an excitation peak electric field strength of 0.6 V nm^{-1}, leading to electrons being emitted from the gap region. The movie shows the temporal evolution of the transmission of probe electrons through the nanoantenna, as it is changed by the cloud of electrons photoemitted from the gap. The snapshots shown in Fig. 2a are taken from this movie.

Supplementary Movie 2 | Ultrafast point-projection electron movie #2

The same plasmonic nanoantenna as in Movie S1 is illuminated with pulses with a slightly higher excitation peak electric field strength of 0.7 V nm^{-1}. The movie shows again the temporal evolution of the transmission of probe electrons. The differential transmission images shown in Fig. 3a are calculated from snapshots of this movie.

References

1. Schmidt S, Piglosiewicz B, Sadiq D, Shirdel J, Lee J S *et al.* Adiabatic nanofocusing on ultrasmooth single-crystalline gold tapers creates a 10-nm-sized light source with few-cycle time resolution. *ACS Nano* 2012; **6:** 6040-6048.
2. Vogelsang J, Robin J, Piglosiewicz B, Manzoni C, Farinello P *et al.* High passive CEP stability from a few-cycle, tunable NOPA-DFG system for observation of CEP-effects in photoemission. *Opt Express* 2014; **22**: 25295-22306.
3. Groß P, Esmann M, Becker S F, Vogelsang J, Talebi N, Lienau C. Plasmonic nanofocusing–grey holes for light. *Advances in Physics: X* 2016; **1**: 297-330.
4. Ropers C, Solli D, Schulz C, Lienau C, Elsaesser T. Localized multiphoton emission of femtosecond electron pulses from metal nanotips. *Phys Rev Lett* 2007; **98**: 043907.
5. Stockman M I. Nanofocusing of optical energy in tapered plasmonic waveguides. *Phys Rev Lett* 2004; **93**: 137404.
6. Vogelsang J, Robin J, Nagy B J, Dombi P, Rosenkranz D *et al.* Ultrafast electron emission from a sharp metal nanotaper driven by adiabatic nanofocusing of surface plasmons. *Nano Lett* 2015; **15**: 4685-4691.
7. Ehberger D, Hammer J, Eisele M, Krüger M, Noe J *et al.* Highly coherent electron beam from a laser-triggered tungsten needle tip. *Phys Rev Lett* 2015; **114**: 227601.
8. Herink G, Solli D, Gulde M, Ropers C. Field-driven photoemission from nanostructures quenches the quiver motion. *Nature* 2012; **483:** 190.
9. Park D J, Piglosiewicz B, Schmidt S, Kollmann H, Mascheck M, Lienau C. Strong field acceleration and steering of ultrafast electron pulses from a sharp metallic nanotip. *Phys Rev Lett* 2012; **109**: 244803.
10. Piglosiewicz B, Schmidt S, Park D J, Vogelsang J, Groß P *et al.* Carrier-envelope phase effects on the strong-field photoemission of electrons from metallic nanostructures. *Nat Photonics* 2014; **8**: 37-42.
11. Latychevskaia T, Longchamp J-N, Escher C, Fink H-W. Holography and coherent diffraction with low-energy electrons: A route towards structural biology at the single molecule level. *Ultramicroscopy* 2015; **159**: 395-402.
12. Latychevskaia T, Longchamp J-N, Fink H-W. When holography meets coherent diffraction imaging. *Opt Express* 2012; **20**: 28871-28892.
13. Longchamp J-N, Rauschenbach S, Abb S, Escher C, Latychevskaia T *et al.* Imaging proteins at the single-molecule level. *Proc Nat Acad Sci USA* 2017; **114**: 1474-1479.
